# Mining candidate genes for maize plant height based on a GWAS, Meta-QTL, and WGCNA

**DOI:** 10.3389/fpls.2025.1587217

**Published:** 2025-06-23

**Authors:** Fu Qian, Zhanqin Zhang, Shubin Chen, Zhiqin Sang, Weihua Li

**Affiliations:** ^1^ The Key Laboratory of Oasis Eco-Agriculture, College of Agriculture, Shihezi University, Shihezi, China; ^2^ Xinjiang Academy of Agricultural and Reclamation Science, Shihezi, China

**Keywords:** maize, plant height, GWAS, WGCNA, Meta-QTL, candidate gene

## Abstract

**Introduction:**

In maize, plant height (PH) is one of the most important agronomic traits that directly influences planting density and yield. Therefore, identifying candidate genes related to PH will help manipulate maize yield indirectly.

**Methods:**

The present research carried out a genome-wide association study (GWAS) of PH using a natural population of 580 maize inbred lines. Further, after collecting the published transcriptome data of maize B73, tissue-specific gene co-expression modules related to PH were generated using weighted gene co-expression network analysis (WGCNA). Furthermore, a meta-analysis of the already reported PH-related quantitative trait loci (QTLs).

**Results:**

The integrated analysis of the results based on the different approaches screened three candidate genes: *Zm00001d031796*, encoding AP2-EREBP transcription factor 172; *Zm00001d009918*, encoding Phytochrome A-associated F-box protein; and *Zm00001d042454*, encoding plastid specific ribosomal protein 4.

## 1 Introduction

Maize (*Zea mays* L.) is a globally important food crop with a wide range of uses. It plays a crucial role in ensuring food security and promoting sustainable agricultural development. Plant height (PH) is one of the most crucial agronomic traits of maize, directly affecting growth development, photosynthetic capacity, planting density, lodging resistance, and final yield. Maintaining optimal PH is essential for achieving high and stable yield in maize. It is also crucial for having an ideal plant architecture, which is conducive to improving plant density and maize yield (Jafari et al., 2024).

Numerous studies have shown that PH in maize is a complex quantitative trait, regulated by multiple genes, and mainly manifested by the co-regulation of main effect genes and micro effect genes (Fei et al., 2022). A study by Yang et al. in maize identified 29 quantitative trait loci (QTLs) related to PH through QTL mapping using bi-parental immortalized heterozygous populations (Yang et al., 2024). Similarly, Zhang et al., based on a genome-wide association study (GWAS) using an F_1_ population consisting of 300 maize hybrids with 17,652 SNPs, identified nine significant SNPs and two candidate genes (*Zm00001d018617* and *Zm00001d023659*) associated with PH (Zhang et al., 2019). Thus, considering the complexity of PH and its sensitivity to various environmental factors, we speculate that identifying candidate genes related to PH and analyzing the genetic basis of PH will provide the basis for breeding improved maize varieties (Zhang et al., 2019).

With the advancement of sequencing technology, GWASs and weighted gene co-expression network analysis (WGCNA) have been used to elucidate the genetic basis of variations in the phenotypic traits of various plant species. Studies have demonstrated the ability of GWASs to uncover marker-trait associations and identify the underlying loci and genes (Han et al., 2024). Meanwhile, WGCNA has been effectively used to investigate gene roles via network diagrams and identify the key genes regulating a trait from transcriptome datasets (Li et al., 2025). Recently, GWASs, linkage mapping, and transcriptome analysis have been integrated to reveal the genetic basis of plant architecture-related traits in maize, predicting that two candidate genes, *Zm00001d044730* and *Zm00001d021574*, were related to maize plant architecture (Lu et al., 2024). Similarly, global transcriptome and WGCNA revealed hybrid-specific modules and candidate genes related to PH in maize (Wang et al., 2018b). Additionally, the generation of gene co-expression modules for PH and ear height by WGCNA revealed the biological functions of the specific modules and identified hub genes within those modules in maize (Li et al., 2019a). Meta-QTL (MQTL) analysis also has emerged as a powerful approach to refine and consolidate data from multiple QTL studies, thereby improving the precision and utility of genetic markers in breeding. In maize, MQTL analysis has been conducted for different traits. For instance, QTLs were identified at five positions for internode length above the uppermost ear using four sets of recombinant inbred line populations in three environments. Genetic maps and initial QTLs were integrated through meta-analyses across the four populations. Of the 70 initial QTLs, 46 were integrated in 14 MQTLs by meta-analysis (Ku et al., 2015). A meta-analysis of 2,974 QTLs associated with 169 component traits revealed 68 MQTLs across diverse genetic backgrounds and environments, unraveling the genetic framework associated with grain quality and yield-related traits in maize (Sethi et al., 2023). A meta-analysis of 917 QTLs associated with root traits in maize predicted 68 MQTLs, the result of which could aid in QTL cloning and pyramiding in developing new maize varieties with specific root architecture for proper plant growth and development under optimum and abiotic stress conditions (Karnatam et al., 2023).

Research has shown that PH is regulated by phytohormone biosynthesis, transport- and signaling-related gene regulation, and other non-hormonal pathways. A few phytohormones, such as gibberellin, brassinolide, auxin, ethylene, and strigolactone, influence PH by regulating cell wall remodeling in maize. Non-hormonal pathways regulate maize PH by altering cortical microtubule arrangement or affecting floral transition, modulating cell elongation and controlling cortical microtubule orientation (Li et al., 2019b). Additionally, the CLAVATA(CLV)-WUSCHEL(WUS) feedback loop is a key pathway regulating the maintenance and development of apical meristems in plants (Somssich et al., 2016). The CLV-WUS feedback loop also regulates PH in maize; this feedback loop plays a decisive role in the morphogenesis of maize PH (Bommert et al., 2013). Although several PH-related genes have been cloned, most mutants of these genes carry deleterious effects. For example, mutations in genes related to meristem fate often change PH, adversely affecting flower development, which is not conducive to high yields (Zhang et al., 2018). Similarly, the mutations in genes related to the hormone pathway in maize shorten the internodes and reduce PH, affecting the development of maize floral organs (Wang et al., 2016). These mutations have also been proven to reduce the biomass of maize, decreasing the supply capacity of the source and the capacity of the sink, which is extremely unfavorable for a high yield of maize and difficult to apply in maize breeding (Jafari et al., 2024; Liu et al., 2020).

Therefore, the present study comprehensively analyzed the maize genetic data at different levels using a GWAS, Meta-QTL analysis, and WGCNA to identify candidate genes associated with PH. This multidimensional approach will provide accurate information on candidate genes, supporting molecular breeding of maize. Additionally, by identifying key genes related to PH, we expect to provide novel insights for breeding maize with high and stable yields.

## 2 Materials and methods

### 2.1 Material and experimental design

A total of 580 maize inbred lines derived from the Canadian early maturing improved group, Iowa Stiff Stalk Synthetic (BSSS), Non-Stiff Stalk (NSS), P group, Huanghuaihai group, European KWS (KWS SAAT SE & Co. KGaA) series, and Pioneer series were used in this study; these lines were provided by the Crop Institute of Xinjiang Academy of Agricultural and Reclamation Sciences. The natural population was planted in Shihezi (44.31° N, 85.99° E) over 3 years (2019, 2020, and 2021), with a planting density of 105,000 plants/ha. The planting was carried out adopting an α Latin square design with two repetitions. Each field was divided into five blocks, and each block consisted of 116 rows, with one maize inbred line grown in a single row (row length of 4.5 m and row spacing of 0.55 m). The fields were managed in accordance with the standard local measures. PH was measured from the soil level to the tip of the main inflorescence. The phenotypic data analysis of PH has been published in previous articles, and detailed information can be obtained from http://www.ymkx.com.cn/jms/article/abstract/20240109.

### 2.2 Genome-wide association study

Genomic DNA was extracted from the fresh leaves of the maize inbred lines using the modified CTAB procedure, and the quality of DNA extraction was tested. SNP genotyping data were obtained using Maize SNPs 40K genotyping by target sequencing (GBTS) technology (Guo et al., 2021; Xu et al., 2020). The SNPs with a minor allele frequency (MAF) > 0.05 and a missing rate < 0.05 were retained using PLINK1.9 software. To eliminate the influence of environmental (year) variation in phenotypic values, a mixed linear model was constructed using the R package lme4 to estimate the best linear unbiased estimate (BLUE) of PH for a subsequent GWAS. The Bayesian information and linkage disequilibrium iteratively nested keyway (BLINK) model in the R package GAPIT was used for the GWAS (Huang et al., 2018). Principal component analysis (PCA) and kinship were used as covariates in the association analysis to reduce false positives. The research results on population structure, PCA, and kinship have been published in previous studies: https://doi.org/10.3390/plants12223806. The Bonferroni correction was used to control the probability of false positives (*p* = 0.05/N, N is the total number of SNPs). The information on the candidate genes was obtained from the MaizeGDB (http://www.maizegdb.org/) genome browser (B73_RefGen_v4) based on the physical positions of the SNPs significantly associated with the target trait and chromosome average linkage disequilibrium (LD) decay distance (*r* ^2^ = 0.1).

### 2.3 Weighted gene co-expression network analysis

The transcriptome data generated for the maize B73 inbred line samples at various developmental stages and from different tissues (leaves, apical meristem, stem, silk, anther, ear, root, seed) were obtained from MaizeGDB (Stelpflug et al., 2016). The WGCNA (v1.72) package in the R software was used to create the WGCNA, with a gene expression matrix derived from the gene expression levels of all the samples. Considering that genes with low expression in all tissues may not be biologically significant, genes with the highest expression fragments per kilobase per million reads (FPKM) value less than five were filtered out (Luo et al., 2022). To ensure that the data conform to the scale-free network distribution, the weighting coefficient *β* value was screened and the network topology was analyzed using the pickSoftThreshold function in the WGCNA package, and the gene co-expression network was constructed based on the value of the threshold parameter *β* when the fitted curve first approached 0.85 (Wang et al., 2023a). The dynamic tree cut algorithm was used to identify the co-expression modules of the transcriptome expression data, and the minimum number of variables to be included in the module (minModuleSize) was set to 30. Modules with a minimum module size of 30 and a merge cut height of 0.25 were merged if their similarity exceeded 0.75. The different tissues were used as traits to create a phenotype matrix, and the correlation coefficients between the module eigengene (ME) and the different organizations were calculated. Among the modules, those with correlation coefficients above 0.65 were defined as tissue-specific modules to identify the biologically significant ones (Downs et al., 2013; Yang et al., 2019). The weight values between different genes within each tissue-specific module were calculated based on the topological overlap matrix. The higher the weight value, the higher the degree of association between the genes. Finally, the top 10% of genes that interact with reported PH genes in the network were selected based on their weight values (Li et al., 2019a), and the regulatory network was visualized using the Cytoscape software.

### 2.4 Collection of QTL information related to the PH of maize

More than 40 articles published from 2006 to 2023 were retrieved from the Web of Science (http://www.webofknowledge.com/) and China National Knowledge Infrastructure (CNKI) (https://www.cnki.net/) using the keywords maize, PH, and QTL (**Supplementary Table S1**). For the experiments with a complete genetic map and QTL information, the information was arranged according to the format required by the software; the information on all essential parameters, such as QTL name, position, linkage group, LOD (logarithm of odds), CI (confidence interval), and phenotypic variance explained (R^2^), was used for the preparation of QTL files.

### 2.5 QTL projection and meta-analysis

The QTL map projection and the following meta-analysis were implemented using the BioMercator4.2.3 software (Sosnowski et al., 2012). The collected maize PH QTLs were iteratively projected onto the target map IBM2_2008 Neighbors, and MQTL analysis was carried out on the QTL clusters present on each chromosome. Then, the best-fitting model was determined based on the minimum Akaike information content (AIC) value. Typically, the model with the lowest AIC represents the most accurate one, and this was used to determine the number of MQTLs. In this analysis, the initial number of QTLs used for the Meta-QTL analysis was never less than two (Kumar et al., 2021). The physical length of the identified meta-QTL was determined and analyzed to retrieve the candidate genes linked with PH from the maizeGDB database.

## 3 Results and analysis

### 3.1 Genome-wide association study

**Figure 1:**
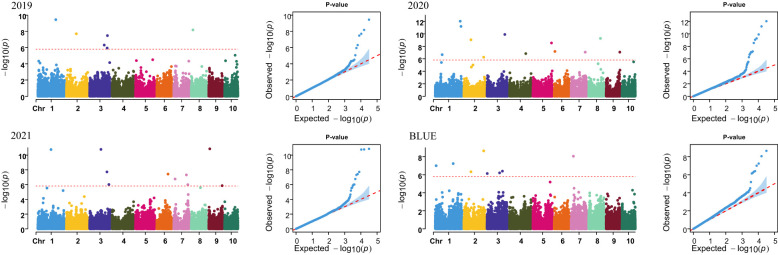
Manhattan plot and QQ plot of PH.

A total of 31,826 high-quality SNPs were retained to conduct a GWAS for PH in maize. The BLINK model in the GAPIT package was used for the GWAS, and PCA and a kinship matrix were used as covariates to control for false positives. Manhattan and QQ plots showed that the false positives for PH were well controlled (**Figure 1**). Then, using *p* = 1.57 × 10^−6^ as the threshold level line for significant correlation to select significant SNPs, 31 SNPs significantly associated with PH were identified in maize in four environments (3 years and BLUE value) (**Supplementary Table S2**).

Further, to enhance and ensure the reliability of these significant SNPs, SNPs that were repeatedly detected in at least two environments were selected for subsequent analysis. There were five SNPs co-localized in different environments, one SNP each was found on chromosomes 1, 2, 3, and 7, while two were localized on chromosome 2, with the phenotypic variance explained ranging from 0.62% to 2.48% (**Table 1**).

**Table 1:** Significant colocalization of SNPs for PH.

SNPs	Chr	Position (bp)	Alleles	PVE (*P*-value)			
				2019	2020	2021	BLUE
1_201909524	1	201909524	G/C	2.48% (3.60E-10)			1.67% (5.74E-08)
3_200267362	3	200267362	C/T	1.31% (3.32E-08)	0.88% (1.24E-10)		
2_79335071	2	79335071	A/G		1.4% (8.81E-10)		1.74% (4.63E-07)
7_157639029	7	157639029	C/T		1.16% (8.53E-08)	0.83% (1.02E-06)	
2_221912962	2	221912962	C/G		0.62% (5.47E-07)		1.18% (2.22E-09)

PH, plant height; Chr, chromosome; PVE, phenotypic variance explained; BLUE, best linear unbiased estimate.

### 3.2 Effect of the allelic variations on PH

**Figure 2:**
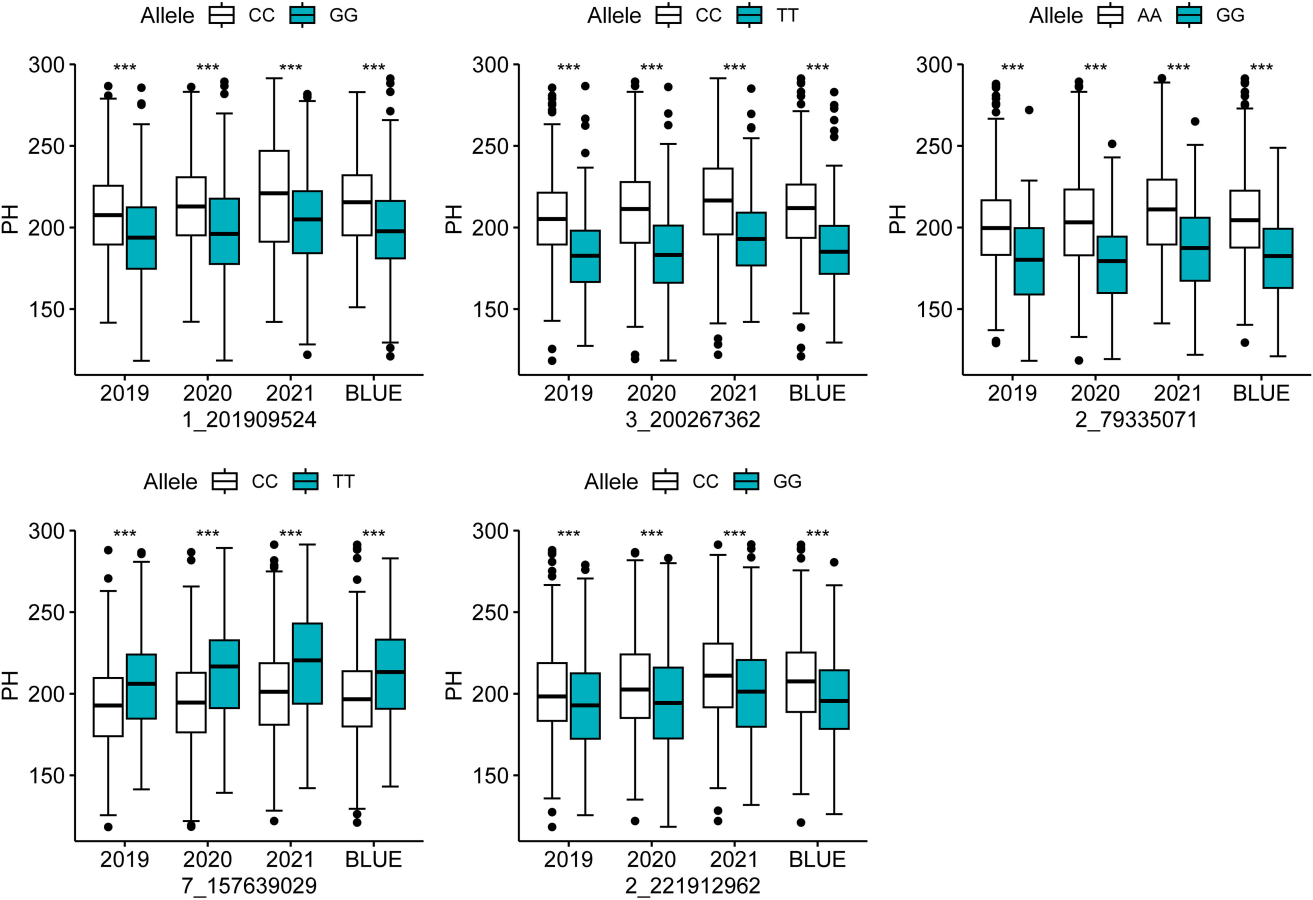
Analysis of the allelic effects of colocalized SNPs for PH. *** indicates a significant difference at the 0.001 probability level.

To test the effect of different alleles of co-located SNPs on PH, a *t*-test was performed. The test revealed that the variations in the alleles of the five co-localized SNPs resulted in highly significant phenotypic differences in PH in all four environments. In the four environments, the SNP 1_201909524 contributed to 14.02–17.53 cm higher PH in the CC genotype than in the GG genotype; the SNP 3_200267362 increased PH of the CC genotype by 21.67–24.41 cm compared with the TT genotype; SNP 2_79335071 increased the PH of the AA genotype by 20.64–25.50 cm compared with the GG genotype; SNP 7_157639029 increased the PH of the TT genotype by 14.10–20.15 cm compared with the CC genotype; SNP 2_221912962 increased the PH of the CC genotype by 8.54–11.78 cm compared with the GG genotype (**Figure 2**; **Supplementary Table S3**). These observations suggest the specific effects of the SNPs on the phenotypic variations in PH, providing valuable information for marker-assisted selection aiming for dwarfness in maize.

### 3.3 Construction of the co-expression network by WGCNA

**Figure 3:**
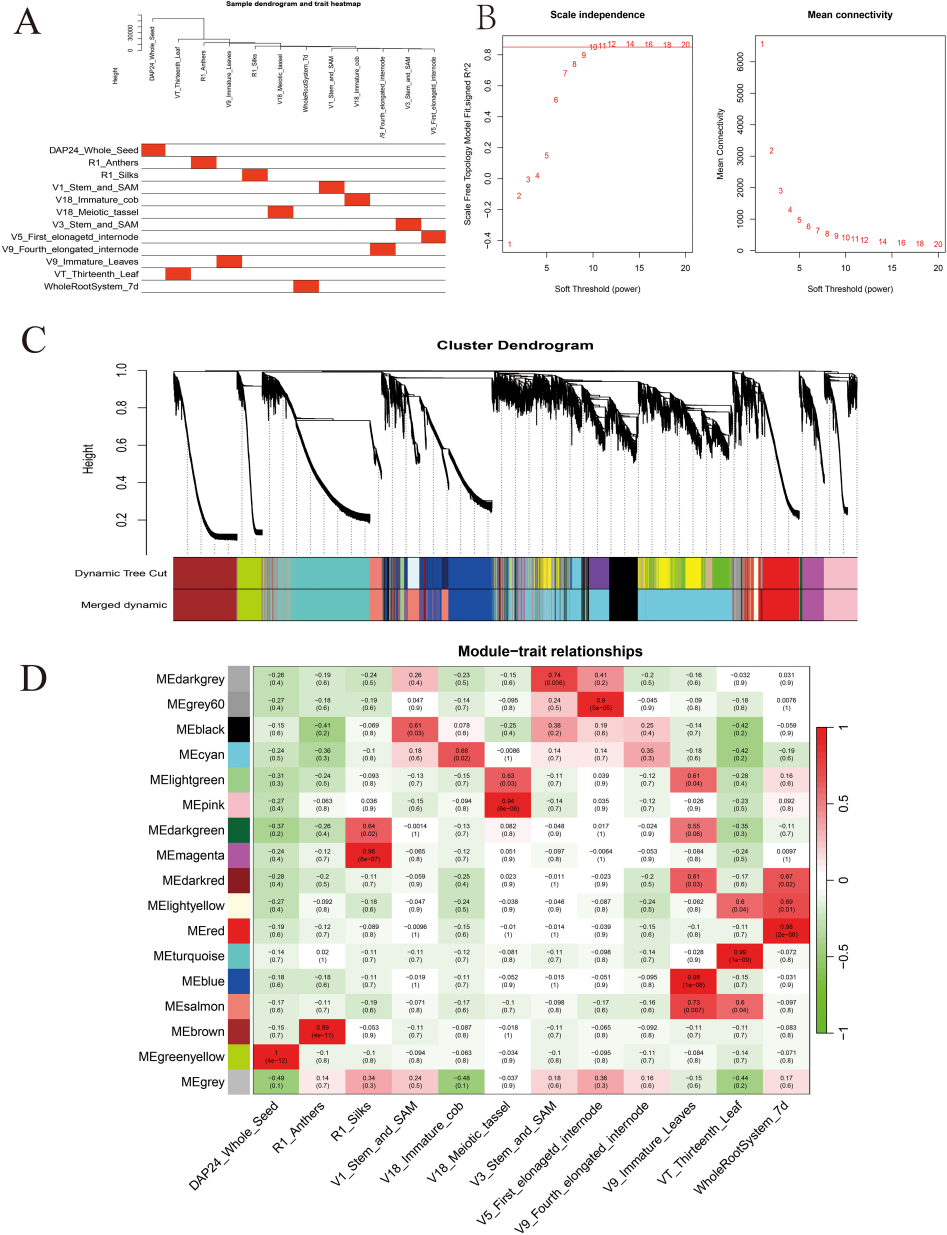
Construction of the co-expression network by weighted gene co-expression network analysis. **(A)** The clustering dendrogram of samples and tissue correspondence; **(B)** the determination of soft threshold; **(C)** gene clustering and module construction; **(D)** correlation between traits and modules. The red color represents a positive correlation between the module and the trait. The green color represents a negative relationship between the module and the trait.

Based on the FPKM values (FPKM>5), 20,548 highly expressed genes were obtained from the maize gene expression matrix. Subsequent sample clustering based on the expression levels of these genes showed that the gene clustering tree for each tissue corresponded well with its respective tissue type (**Figure 3A**). Based on the soft threshold calculation results, *β*=10 was chosen for network construction, and the dynamic pruning tree method was used to merge modules with similar expression levels (**Figures 3B, C**). This approach generated 17 co-expression modules, represented by different colors; the Cyan module had the most genes and Gray represents genes that cannot be classified into any module (**Figure 3C**).

A detailed analysis revealed that 13 out of the 17 modules were highly specific to tissues, and most tissues had modules that were highly correlated with them. The Darkgrey module was significantly correlated with V3_Stem_and_SAM (r=0.74, *P*=0.006), the Grey60 module was significantly correlated with V5_First_elonagetd_internode (r=0.90, *P*=5e-05), the Blue and Salmon modules were significantly correlated with V9_Immature_Leaves (r=0.98, *P*=1e-08) (r=0.73, *P*=0.007), and the Turquoise module was significantly correlated with VT_Thirteenth_Leaf (r=0.99, *P*=1e-09) (**Figure 3D**). Studies have proven that the shoot apical meristem, internodes, leaves and other tissues are closely related to maize stalk growth and development and PH (Wang et al., 2018a). A few researchers have found functional genes for PH, among which one (*D3*) was found in the Grey60 module, four (*Br2*, *NA1*, *RS2*, *ZmACS7*) in the Blue module, two (*D8* and *D9*) in the Salmon module, and seven (*ZmCT2*, *ZmTD1*, *SXD1*, *ELM1*, *ZmBELL10*, *ZmCOP1*, and *ZmCYP90D1*) in the Turquoise module. Therefore, this study focused on these four modules and utilized the reported PH genes of these modules as the hub genes to construct a gene interaction network and screen the candidate genes related to PH.

### 3.4 Meta-QTL analysis

Analysis of almost 40 published articles helped us collect 362 PH-related QTLs that met the predefined criteria (**Supplementary Table S1**). These QTLs were unevenly distributed on each chromosome, ranging from 22 to 66 per chromosome.

**Figure 4:**
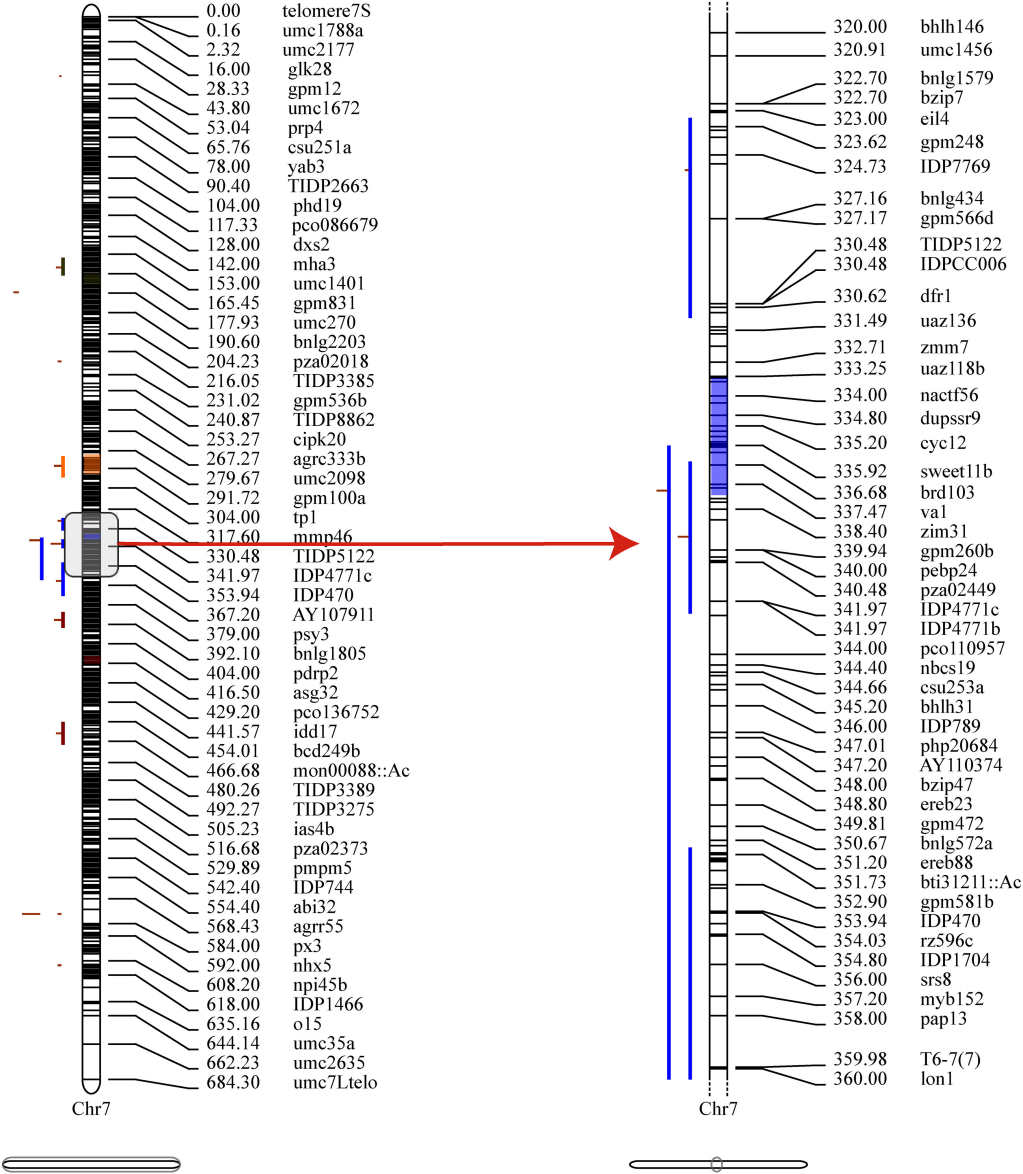
Projection and distribution of QTLs and MQTLs (Meta-QTLs) identified for PH on chromosome 7. The bars on the left side of the chromosome correspond to QTLs related to the plant height trait, the black bars within the chromosomes represent marker density, the colored segments within the chromosome represent MQTLs, and on the right side of the chromosome are molecular markers and genetic distances (cM).

Then, to extract information relevant to the current research, the collected QTLs were projected onto a published reference map, IBM2_2008 Neighbors. This approach identified 18 MQTLs related to PH based on models with the lowest AIC values, and each MQTL contained two to six initial QTLs. No MQTL was detected on chromosomes 2, 4, and 9 (**Supplementary Figure S1**). The CI (95%) of the detected MQTLs varied from 0.43 to 13.36 cM, with an average of 5.14 cM (**Figure 4**, **Supplementary Table S4**).

**Figure 5:**
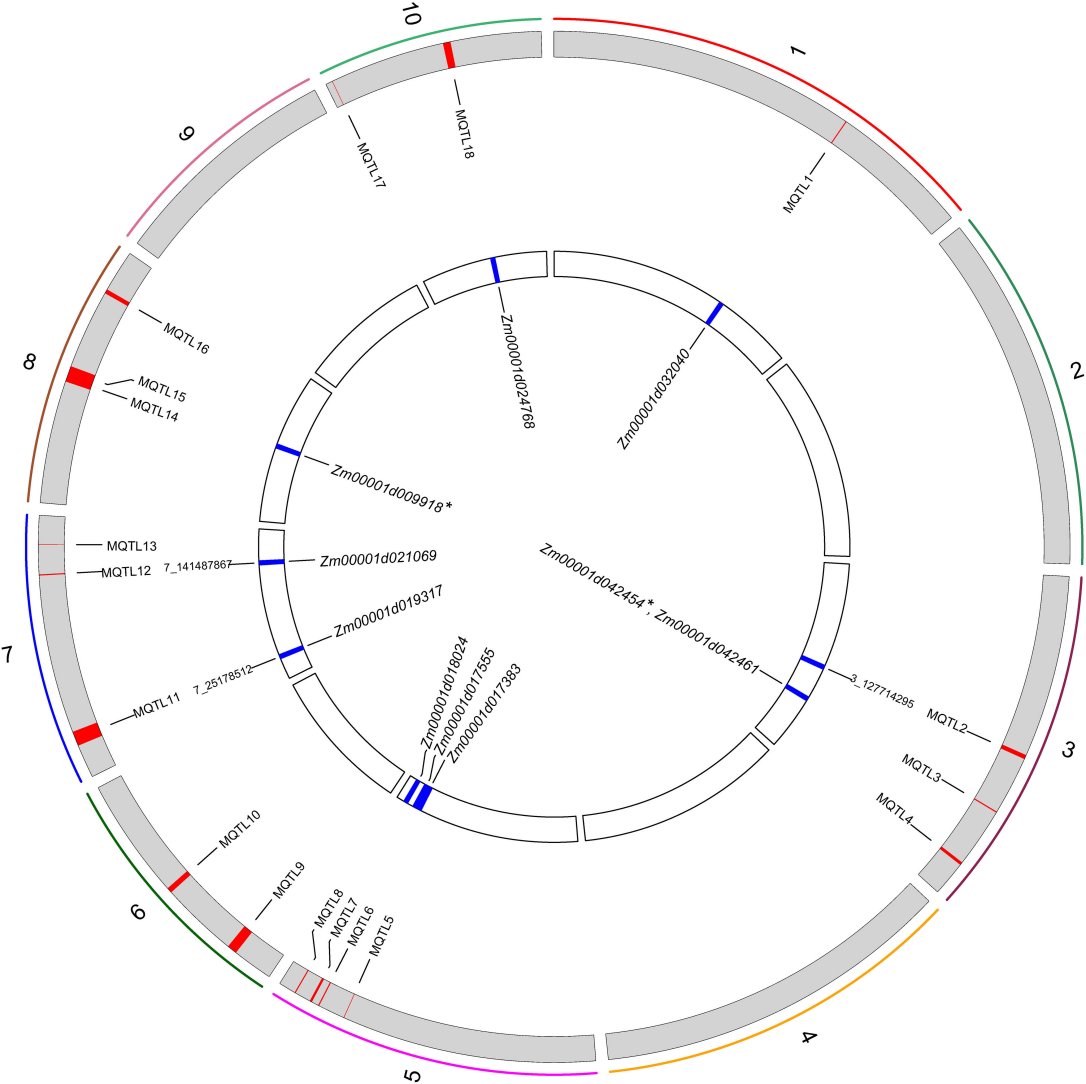
Circos plot showing the Meta-QTLs, significant SNPs, and genes on a physical map. The colored bars show the 10 maize chromosomes, and the red and blue areas represent the positions of MQTL and SNPs on the chromosome. * indicates candidate genes jointly identified by Meta-QTL analysis and WGCNA.

Further, the 31 PH-related SNPs detected by the GWAS in this study were compared with the physical coordinates of the 18 MQTLs. The results showed that two of the GWAS-detected SNPs were located within the intervals of the MQTLs, such as SNP 3_127714295 in the MQTL2 interval and SNP 7_25178512 in the MQTL11 interval. Though SNP 7_141487867 was not found within any MQTL interval, it was very close to MQTL12 (229 kb) (**Figure 5**). These results confirmed the accuracy of the SNPs related to PH identified based on the GWAS.

### 3.5 Identification of candidate genes

Finally, the candidate genes were searched in the B73_RefGen_v4 reference genome based on the information on co-localized SNPs and the LD decay distance of 440 kb (r^2^ = 0.1) in this population. The approach detected a total of 54 genes in the candidate region, and subsequent gene function annotation screened four candidate genes related to PH (**Supplementary Tables S5**, **S6**).

**Figure 6:**
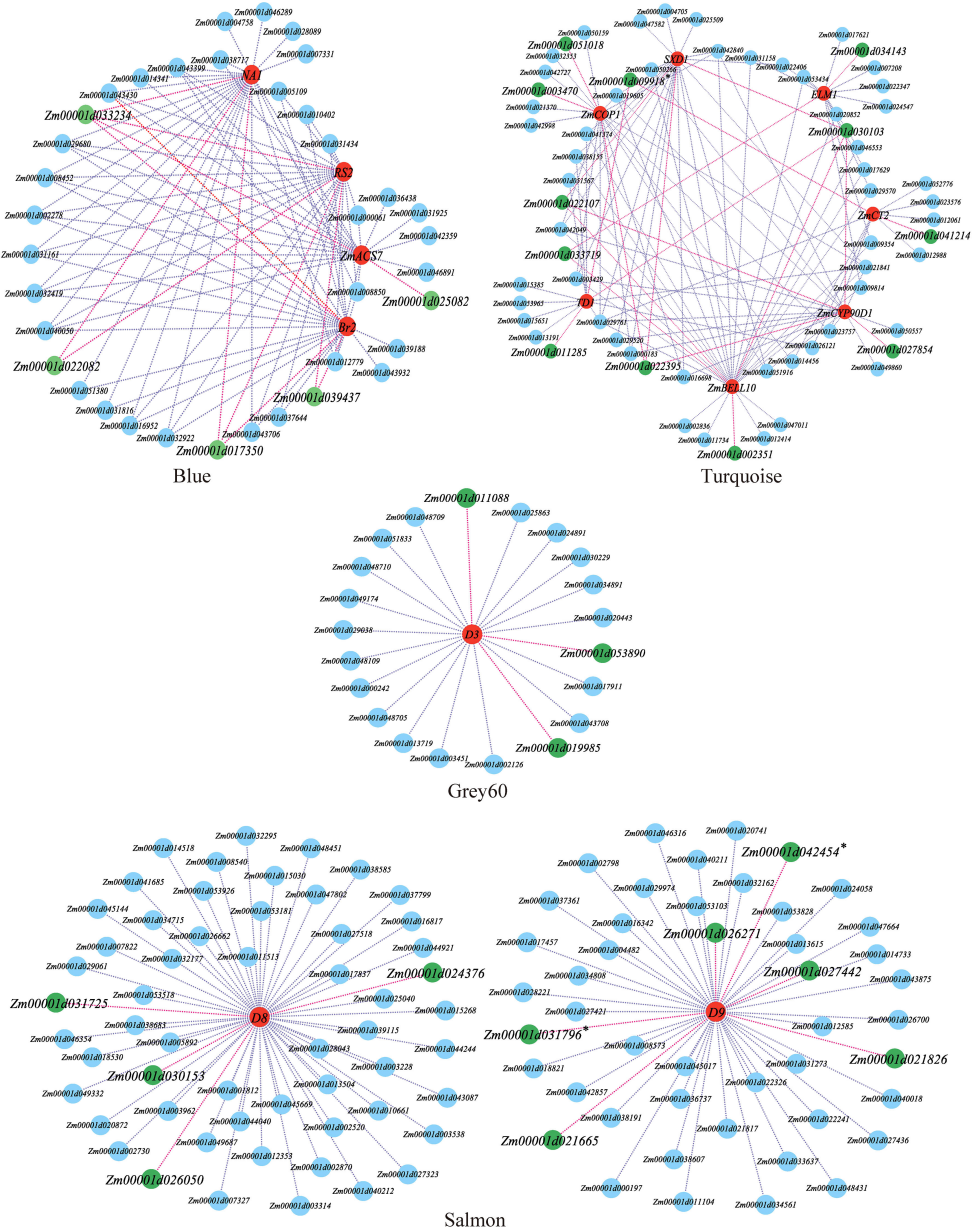
Local regulation network of gene co-expression of key modules. The red nodes are reported plant height trait genes, the green nodes are plant height trait candidate genes. * candidate genes jointly identified by WGCNA and Meta QTL or GWAS.

In the WGCNA, the Turquoise module used seven reported genes to screen 12 candidate genes for PH, the Blue module used four reported genes to screen five candidate genes, the Grey60 module used one reported gene to screen three candidate genes, and the Salmon module used two reported genes to screen 10 candidate genes (**Figure 6**).

Using the positions of the genetic markers at both ends of each MQTL on the B73 genome (B73_RefGen_v4), a total of 150 genes were identified in these MQTL regions (**Supplementary Table S4**). Gene function annotation screened 10 candidate genes associated with PH from nine MQTLs (**Figure 5**; **Supplementary Table S6**).

Thus, in this study, the GWAS and WGCNA jointly arrived at one candidate gene, *Zm00001d031796*, while the WGCNA and meta-analysis jointly mined two candidate genes, *Zm00001d009918* and *Zm00001d042454*. However, the GWAS and meta-analysis did not jointly mine any candidate genes (**Figures 5**, **6**). Finally, no candidate gene was identified by combining the results of all three approaches.

## 4 Discussion

### 4.1 Genetic basis of PH in maize

PH is a crucial factor that determines the architectural composition and influences the yield in maize. An optimal plant architecture ensures a stable and improved yield, as it is a typical polygenic trait in maize that is affected by environmental changes, highly sensitive to the environment, and has a complex genetic basis (Yang et al., 2024). The present study detected many significant SNPs for PH by GWAS in the maize population during different years, but identified fewer SNPs co-localized in multiple environments, mainly because PH is a quantitative trait controlled by multiple genes. Moreover, the environment greatly affects PH. Thus, the different environmental conditions across the 3 years probably led to the differential expression of genes controlling the trait (He et al., 2017). Typically, those detected under multiple environments are stable expression genes, so there were relatively few co-localized SNPs in multiple environments. This result is common in the study of complex traits in maize, as Lu et al. detected 12 SNPs significantly associated with PH in two environments, but did not find co-localized SNPs (Lu et al., 2020). Ma et al. detected 35 SNPs significantly associated with PH in four environments, with only eight co-located SNPs in multiple environments (Ma et al., 2023). These observations indicate that the genetic basis of PH in maize is complex and highly influenced by the environment, and the genetic mechanisms that underlie maize PH diversity are still largely unknown (Wang et al., 2023b). Moreover, the study detected low phenotypic variance explained by the five co-localized SNPs (0.62% to 2.48%), which is expected for a complex trait. Similarly, Ma et al. detected a phenotype contribution rate of only 0.02% to 6.23% (Ma et al., 2023). This is primarily because PH is a quantitative trait controlled by minor genes.

### 4.2 Joint analysis of the GWAS, Meta-QTL analysis, and WGCNA

The present study used a GWAS, Meta-QTL analysis, and WGCNA to analyze the genetic basis of maize PH from genomics and transcriptomics. The combined use of three methods helped us to rapidly identify the genetic intervals and mine the candidate genes (Lu et al., 2024). GWASs have been widely used to reveal the genetic basis of variations in maize phenotypes (Sahito et al., 2024), but they also have certain limitations. For instance, PH, flowering, yield, and many other complex agronomic traits are highly correlated with the population structure, thus, using a GWAS to identify the genes regulating complex agronomic traits affected by the environment is easily influenced by population structure (Camus-Kulandaivelu et al., 2006), and GWASs have insufficient ability to detect quantitative traits controlled by some micro effect multi genes, making it difficult to accurately identify the micro effect locus or genes. In the analysis process, false positive or false negative results may also be introduced, which may lead to inaccurate experimental results.

Despite many QTLs related to PH having been identified in maize, very few have been useful in genetic improvement programs due to their minor effects and the influence of the environment. In such situations, an alternative method or an integrated approach is necessary. A meta-analysis of QTLs integrates information from numerous QTLs across different experiments and populations, narrows the confidence interval, and improves the accuracy of QTL mapping (Goffinet and Gerber, 2000). Although the meta-analysis of QTLs reduces the CI of the original QTL, the MQTL interval still contains a large number of genes. Meanwhile, WGCNA is an effective method to analyze gene co-expression networks and is capable of specifically screening out co-expression modules with high biological significance to the target trait. This approach compensates for the insufficient ability of a GWAS to analyze complex traits and has been proven effective in data mining in various plants (Li et al., 2023). Therefore, combining WGCNA with a GWAS and Meta-QTL analysis will greatly improve the effectiveness of candidate gene mining for PH.

Currently, the integrated analysis of multi-omics is an efficient and popular approach used to mine genes associated with crucial agronomic traits. Multi-omics analysis has been widely used, but most studies have been done using two methods, such as a joint GWAS and WGCNA to uncover the genetic control of calcium accumulation under salt treatment in maize seedlings (Liang et al., 2021). Meta-analysis and WGCNA have revealed the hub genes for seed storage composition during seed development in soybean (Qi et al., 2018). Meta-QTL analysis and a GWAS were used to discover the genomic regions and candidate genes for yield and yield-related traits in bread wheat (Yang et al., 2021). A joint analysis using a GWAS, WGCNA, and Meta-QTL analysis is scarce in the literature. Therefore, the present study employed a GWAS, Meta-QTL analysis, and WGCNA and performed an integrated analysis of the results to mine candidate genes for maize PH. However, common genes were found only between two methods and not among all three. The GWAS and WGCNA jointly mined one candidate gene, while the WGCNA and meta-analysis jointly mined two candidate genes. No common candidate gene was mined based on the results of all three methods, probably due to the relatively small number and low density of SNP markers (31,826) used in this research after the GWAS quality control. It is also possible that although the natural population has been planted for 3 years, it is growing in almost the same environment. PH is a trait influenced by gene-environment interactions, and a GWAS in a single environment may miss gene-environment interactions revealed in other environments, resulting in incomplete gene mining and fewer candidate genes (Hudson et al., 2022). On the other hand, it may be due to the influence of different genetic maps and molecular marker types in QTL studies, resulting in a limited number of available QTLs collected, leading to the inability to detect some related regions and candidate genes (Kaur et al., 2023). GWASs and Meta-QTL analysis use molecular markers and gene loci to mine candidate genes, while WGCNA uses gene expression to mine candidate genes. Different experimental materials and treatments can result in inconsistent gene expression, leading to candidate genes that are not identified consistently across all methods. Although a GWAS, Meta-QTL analysis, and WGCNA were used in this study, the genetic analysis of maize PH from genomics and transcriptomics remains limited. Therefore, genomics, transcriptomics, proteomics, metabolomics, and other omics should be integrated to efficiently analyze the genetic basis of maize and construct a more detailed and complete molecular regulatory network for important maize traits (Han et al., 2022).

### 4.3 Functional prediction of candidate genes associated with PH in maize

The gene *Zm00001d031796* encodes AP2-EREBP transcription factor 172. Apetala2/ethylene response factor is one of the largest families of transcription factors, regulating growth, development, and stress response in plants. In addition to directly regulating genes involved in plant development and stress response, AP2/ERFs mediate signaling of hormones, including stress-associated (abscisic acid and ethylene) and growth-related (gibberellic acid, cytokinin, and brassinosteroid) hormones (Qi et al., 2023). AP2/EREBP-TFs also control various developmental processes. Deregulated expression of AP2/EREBPs caused pleiotropic effects such as decreased cell size, PH, hypocotyl elongation, and fertility in maize (Dietz et al., 2010). Recently, Wang et al. identified *ZmEREB92* and *ZmEREB93* as key candidate genes that regulate ear height and the ratio of ear to PH (Wang et al., 2022).

*Zm00001d009918* encodes a phytochrome A-associated F-box protein. Phytochrome A (PHYA) is the unique far-red light receptor in plants, which precisely regulates the transcription network via multiple pathways. PHYA affects physiological processes such as shade avoidance response, flowering time, plant architecture, and apical dominance in plants (Lei et al., 2024). *ZmphyA* plays an important role in regulating maize PH (Cao et al., 2024). The homologous genes in *Arabidopsis* (*AT4G02440*) encode the F-box protein empfindlicher im dunkelroten licht 1(*EID1*), which negatively regulates phytochrome A (phyA)-specific light signaling. *EID1* also regulates hypocotyl elongation (Marrocco et al., 2006; Zhou et al., 2002) and functions during the shade avoidance response in *Arabidopsis thaliana* by repressing *PHYA* action and thereby allowing seedlings to elongate in the shade (Staudt et al., 2023).

*Zm00001d042454* encodes plastid-specific ribosomal protein 4 (PSRP4). The plant plastids possess a small set of proteins unique to the plastid ribosome, named plastid-specific ribosomal proteins (PSRPs). It has been proposed that these proteins may represent accessory proteins involved in translational regulation (Tiller et al., 2011; Xu et al., 2013). In a study on plant structure using 10 maize recombinant inbred line populations, a new PH QTL, *qPH3*, was fine-mapped to a 600 kb genomic region with three candidate genes, including *Zm00001d042454* (Pan et al., 2017). This research suggests that *Zm00001d042454* plays a role in regulating plant structure, including PH.

Although our study identified several promising candidate genes potentially involved in PH regulation, their functional roles remain to be experimentally validated. One of the limitations of this study is the relatively low marker density (31,826 SNPs) derived from SNP chip genotyping, which restricts the resolution for haplotype analysis and fine mapping of associated genomic regions. Additionally, the lack of whole-genome resequencing data limits our ability to perform comprehensive candidate gene association studies or accurate haplotype reconstruction. To overcome these limitations within the scope of the available data, we performed a haplotype analysis of five colocalized SNPs identified by the GWAS within the LD decay region (**Supplementary Figure S2**). This analysis aimed to characterize the patterns of genetic variation and potential functional combinations among these SNPs. Our long-term objective is to perform whole-genome resequencing on the studied materials to enable more detailed genetic dissection of the loci identified in this study. Future functional analyses should include investigations into the candidate gene expression patterns, subcellular localization, genetic complementation, overexpression, CRISPR/Cas9 vector construction, and genetic transformation. These analyses will help reveal their biological functions and elucidate the genetic mechanisms underlying PH regulation. In other words, the functional validation of key candidate genes will provide novel insights into the genetic and molecular basis of PH in maize.

## 5 Conclusions

The present study detected five significantly co-located SNPs in multiple environments through a GWAS, providing important genetic loci for molecular marker-assisted selection breeding. Three candidate genes jointly mined by multiple methods provide a reference and basis for the precise localization and cloning of genes related to PH. These research results provide valuable information for analyzing the genetic basis of maize PH.

## Data Availability

The datasets presented in this study can be found in online repositories. The names of the repository/repositories and accession number(s) can be found in the article/**Supplementary Material**.
