# Supplementary material for: Mining candidate genes for maize plant height based on a GWAS, Meta-QTL, and WGCNA
Source: Front Plant Sci. 2025 Jun 23;16:1587217. doi: 10.3389/fpls.2025.1587217 (PMC12230025; doi:10.3389/fpls.2025.1587217)
Supplement: Supplementary file 1 [file DataSheet1.docx]

**Supplementary Figure 1.** Projection and distribution of QTL and MQTL (Meta-QTL) identified for plant height trait. Bars on the left side of the chromosome correspond to QTL related to plant height trait, black bars within chromosomes represent marker density, colored segments within the chromosome represent MQTL, and on the right side of the chromosome are molecular markers and genetic distances (cM).


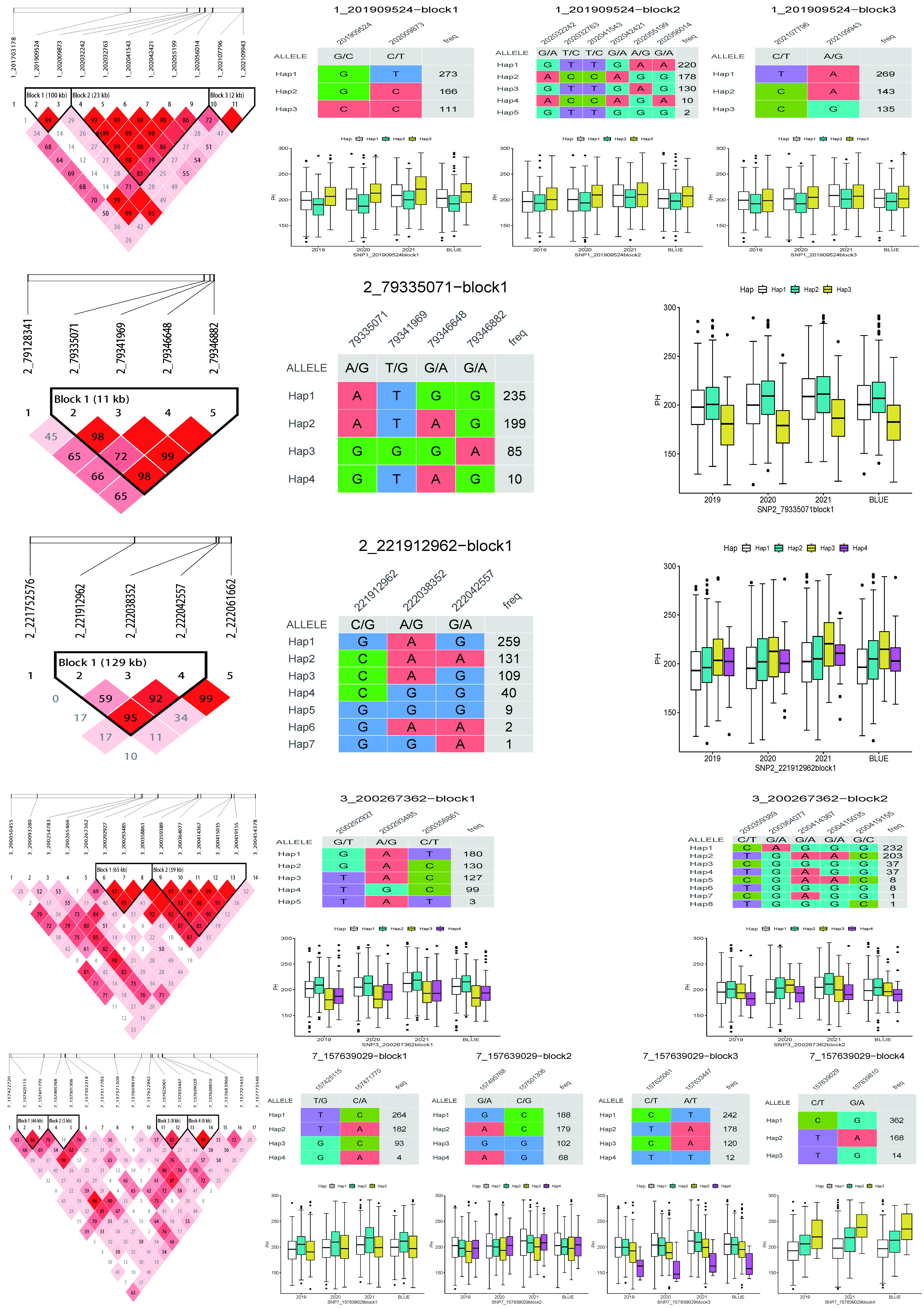


**Supplementary Figure 2.** Haplotype analysis of five colocalized SNPs identified by GWAS within the LD decay region. Note: Only haplotypes with a sample size >10 were subjected to statistical comparison for PH.
